# Physicochemical Properties of Bovine Serum Albumin-Glucose and Bovine Serum Albumin-Mannose Conjugates Prepared by Pulsed Electric Fields Treatment

**DOI:** 10.3390/molecules23030570

**Published:** 2018-03-03

**Authors:** Wenjie Jian, Liangyu Wang, Lanlan Wu, Yuan-ming Sun

**Affiliations:** 1 Institute of Nutrition and food Safety, Xiamen Medical College, Xiamen 361023, China; lanlan_0519@163.com; 2 College of Food Science, South China Agricultural University, Guangzhou 510642, China; 3 Department of Biology and Chemistry Engineering, Fuqing Branch of Fujian Normal University, Fuzhou 50300, China; wly.h@163.com

**Keywords:** bovine serum albumin, glucose, mannose, physicochemical properties, pulsed electric fields treatment

## Abstract

The pulsed electric fields (PEF) treatment is a novel method for obtaining glycated proteins by way of a Maillard reaction between proteins and polysaccharides but its effect on the preparation of protein–monosaccharide conjugate has not been explored. This study aimed to prepare bovine serum albumin (BSA)–glucose and BSA–mannose conjugates using PEF in pH 10.0 at an intensity of 10 or 20 kV/cm, frequency of 1 kHz, pulse width of 20 μs and 73.5 pulses. The conjugates were evaluated for physicochemical properties. The results indicated that PEF not only promoted Maillard reaction between BSA and glucose or mannose but also alleviated the undesirable browning. PEF treatment favored the increased surface hydrophobicity and emulsifying activity in BSA but reduced surface hydrophobicity and foaming stability and improved foaming capacity in BSA–glucose and BSA–mannose conjugates. These findings provided useful considerations in the application of PEF treatment as a potential method to prepare BSA–monosaccharide conjugates by Maillard reaction.

## 1. Introduction

Maillard reaction, a natural reaction occurring between the ε-amino groups in proteins and the reducing carbonyl group in saccharine, is an important method to improve the functionalities of proteins [1]. A lot of improvements have been achieved in the emulsifying ability and thermal stability of proteins after glycation with saccharine via Maillard reaction [2,3]. For instance, excellent emulsifying ability and dispersion stability are usually observed in the glycated proteins [4,5].

The factors influencing the Maillard reaction include food composition, reaction time, temperature, pH and the content and type of saccharine [6]. Monosaccharides have a higher yield and require milder reaction conditions compared with polysaccharides when glycated with proteins [7,8]. Moreover, the type of monosaccharide is also important in glycation [9]. For example, glucose and mannose, common aldose sugars widely used in the food industry, showed a significantly different effect on the glycated bovine serum albumin (BSA), as demonstrated in a previous study using thermal treatment [10].

Thermal treatment, a traditional processing method, not only promotes Maillard reaction but also produces some negative effects [11]. For example, higher temperature or longer incubation time of Maillard reaction might result in the undesirable browning and important physiochemical and structural changes in proteins [12,13]. Furthermore, a carcinogenic compound in humans, acrylamide can be generated during thermal treatment as a result of Maillard reaction. Researches shown that asparagine and its residue in protein is a crucial participant in the production of acrylamide [14]. It was difficult to completely prevent the formation of acrylamide during thermal treatment [15]. These pose challenges for food safety and should be avoided by applying new processing technologies. 

Pulsed electric fields (PEF) treatment, a non-thermal food-processing method, was widely used in killing pathogenic microorganisms and inactivating enzymes [16,17]. As PEF has the characteristics of low heat generation and short treatment time (in milliseconds), it could minimize the loss of color, taste, nutrients and heat-labile functional components of foods [18]. It was successfully applied in the liquid food for pasteurization or enhancing processing instead of the traditional thermal treatments [19]. Thus, PEF may have a potential positive effect in enhancing Maillard reaction. So far, a few reports are available on the application of PEF on the Maillard reaction. An accelerated Maillard reaction was observed in the preparation of BSA–dextran [20] and whey protein isolate–dextran conjugate [21] when the electric field intensity was higher than 10 kV/cm. Besides, PEF was also found to promote the Maillard reaction in an asparagine–glucose and glycine–glucose solution at higher electric field intensity [22]. These results demonstrated that PEF was a method to promote Maillard-based copolymerization or glycation and signaled that glycation by PEF might be a good choice to improve the functional properties of proteins, without or reducing the negative effect induced by glycation via thermal treatments. However, the reports about the effects of PEF on proteins and their glycation are still scarce and the physicochemical properties of glycated proteins prepared by PEF have not been fully explored. 

BSA, an important ingredient and a desirable model protein for food systems, is generally used in the study of Maillard-based glycation [23]. Besides, BSA is a by-product from slaughterhouses. Its usage and value-added processing could benefit reducing industrial wastes and valorizing the price. So, BSA was used in this research. Glucose and mannose showed significantly different effects on the glycation of BSA prepared by thermal treatment, as found in a previous study [10]. Therefore, this study aimed to obtain BSA–glucose and BSA–mannose conjugates at pH 10.0 and the electric field intensity of 10 kV/cm and 20 kV/cm, respectively. As widely known that pH played an important part in Maillard reaction and alkaline condition (pH 10.0) favored the improvement on reaction extent [6,22]. The focus of this study is the effect of PEF on the glycation of BSA. Thus, the value of pH was set at pH10.0 and other pH values were neglected. The physicochemical properties of BSA, BSA–glucose and BSA–mannose conjugates were fully examined. Consequently, the effect of PEF on the glycation of BSA was better understood. Despite of the report that alkaline suspension could produce foam, no significant foaming was observed during PEF. This may be jointly ascribed to the differences in conditions, such as temperature, concentration of protein, etc. Hence, the blank experiment about foaming activity of BSA in pH10.0 was not included.

## 2. Results and Discussion

### 2.1. Changes in A294 and Browning Intensity

**Figure 1:**
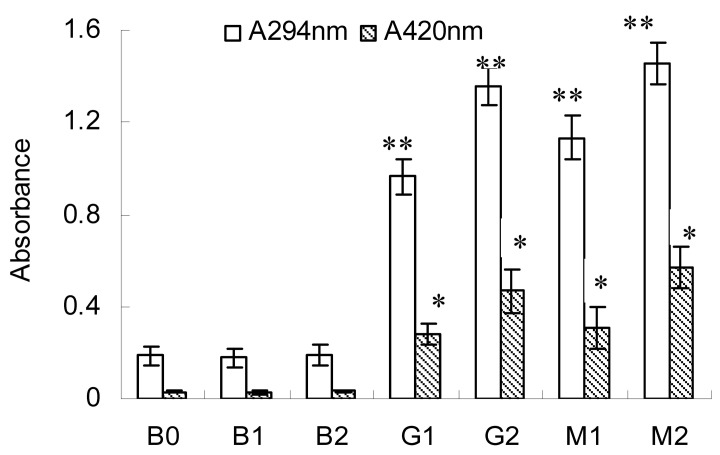
Absorbance measured at 294 and 420 nm of BSA, BSA–glucose and BSA–mannose at intensities 10 and 20 kV/cm. Data are mean ± standard deviation, *n* = 3. * *p* < 0.05, ** *p* < 0.01 versus control (B0).

The changes in the UV absorbance value at 294 nm (A_294_) and browning intensity (A_420_) are shown in Figure 1. A_294_ was the indicator for the amount of intermediate products of Maillard reaction and A_420_ indicated the extent to which the Maillard reaction took place in foods [24]. Both A_294_ and A_420_ changed significantly (*p* < 0.05) in BSA–glucose (G1 and G2) and BSA–mannose (M1 and M2) conjugates at intensities 10 and 20 kV/cm. In contrast, no significant changes were observed in the blank test (B1 and B2) regardless of the PEF intensities applied. Besides, the values of A_420_ in BSA–glucose (G1 and G2) and BSA–mannose (M1 and M2) conjugates were much lower than those in a previous study using thermal treatment [10]. This fully demonstrated that preparation by PEF treatment could alleviate the browning intensity of Maillard reaction.

Furthermore, higher values of A_294_ and A_420_ were observed at the intensity of 20 kV/cm (G2 and M2) compared with those at the intensity of 10 kV/cm (G1 and M1). This observation demonstrated that more intermediate and final products of Maillard reaction were produced at the intensity of 20 kV/cm. This was in accordance with the report in previous studies that higher PEF intensity favored the promotion of Maillard reaction [22]. Moreover, higher values of A_294_ and A_420_ were found in BSA–mannose (M2 and M1) conjugate compared with those in BSA–glucose (G2 and G1) conjugate at the same PEF intensities applied. This finding further confirmed the previous results, that is, mannose displayed a higher reaction extent compared with glucose when glycated with BSA under the same conditions [10]. This difference in reaction extent between glucose and mannose was ascribed to their position of the ring opening equilibrium (stereochemistry of hydroxyl in C3) [25].

### 2.2. Conjugation Ratio in Glycated-BSA Conjugates

**Figure 2:**
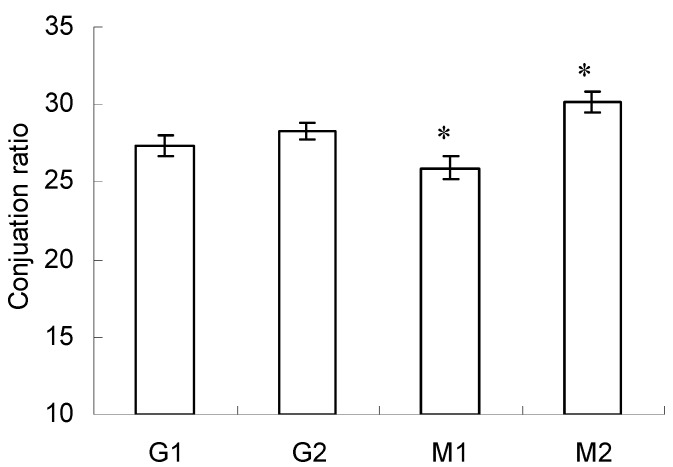
Conjugation ratio of BSA–glucose and BSA–mannose at intensities 10 and 20 kV/cm. Data are mean ± standard deviation, *n* = 3. * *p* < 0.05 versus control (G1).

Besides the aforementioned A_294_ and A_420_, conjugation ratio in glycated proteins was also generally used to quantify the exact extent of Maillard reaction. The higher conjugation ratio meant more reducing sugar or polysaccharides attached to proteins [26]. The conjugation ratio of BSA–glucose was 27.29 ± 0.68 (G1) and 28.26 ± 0.56 (G2), at the intensity 10 or 20 kV/cm (Figure 2). No significant difference was observed in terms of the effect of PEF intensity on conjugation ratio of BSA–glucose.

Nevertheless, the PEF intensity was associated with the conjugation ratio in BSA–mannose (*p* < 0.05). The conjugation ratio of BSA–mannose was 25.92 ± 0.77 (M1) and 30.11 ± 0.63 (M2), at the intensity 10 or 20 kV/cm. The result indicated that the graft reaction between BSA and mannose took place more effectively at higher PEF intensity, as evidenced by the higher conjugation ratio. Such distinct differences in conjugation ratio observed between glucose and mannose might be due to their chemical structure [25] and the detailed mechanisms should be further analyzed in the future.

Moreover, the conjugation ratios observed in this study were much higher than those reported in a previous study using thermal treatment [10]. This indicated that PEF at proper intensities promoted Maillard reaction, as found in previous studies [18].

### 2.3. Emission Fluorescence Spectroscopic Analysis

The intrinsic fluorescence spectrum was a useful tool to measure the surface hydrophobicity in BSA by elucidating the conformational change around the tryptophan residues. The fluorescence emission maximum (*λ*_max_) moved to a higher wavelength (red shift) when tryptophan residues were exposed more to the solvent. Further, the fluorescent intensity decreased if tryptophan residues interacted with some quenching agents in proteins or solvent [5].

**Figure 3:**
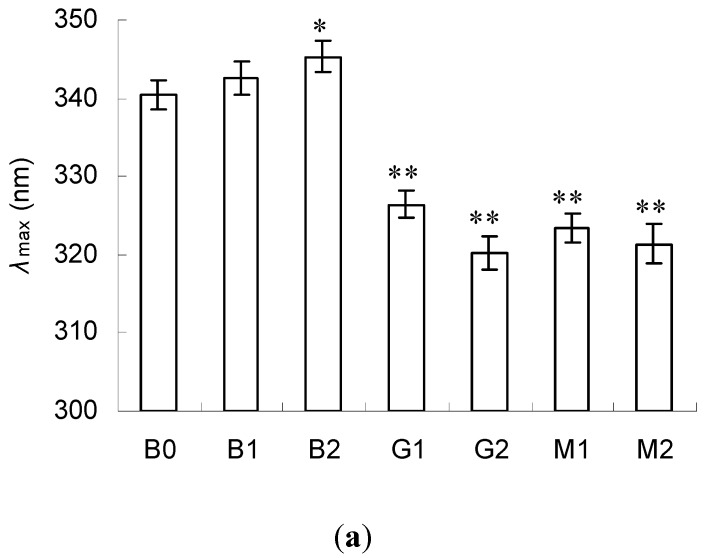

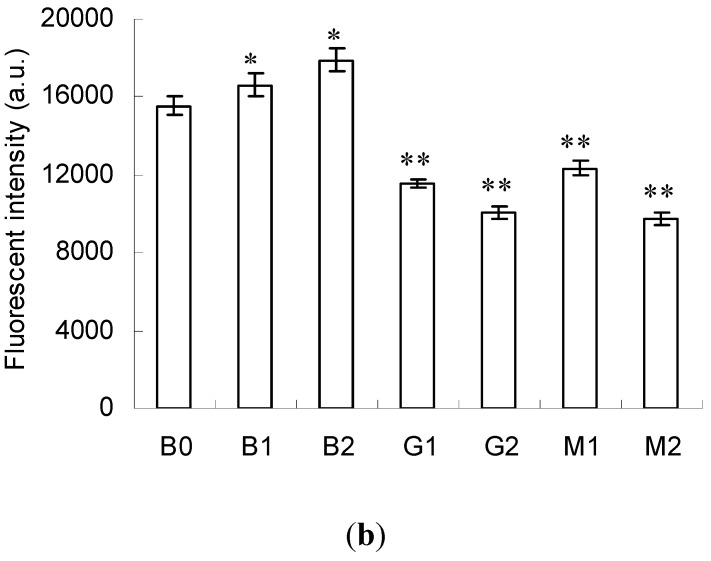
Fluorescence emission maximum (**a**) and fluorescence intensity (**b**) of BSA, BSA–glucose and BSA–mannose at intensities 10 and 20 kV/cm. Data are mean ± standard deviation, *n* = 3. * *p* < 0.05, ** *p* < 0.01 versus control (B0).

The fluorescence emission spectra of BSA, BSA–glucose and BSA–mannose were measured (Figure 3). When excited at 280 nm, BSA exhibited a fluorescence emission maximum (*λ*_max_) at 340 nm (B0). The treatment of PEF at the intensity of 10 kV/cm had no effect on *λ*_max_ and fluorescent intensity (B1). However, a marked red shift and increased fluorescent intensity (*p* < 0.05) were observed when BSA was treated at the intensity of 20 kV/cm (B2). This demonstrated that PEF treatment at proper intensity exposed tryptophan residues more to the solvent and strengthened the surface hydrophobicity of BSA [27].

In contrast, marked blue shift and decreased fluorescent intensity (*p* < 0.05) were found in BSA–glucose (G1 and G2) and BSA–mannose (M1 and M2) at intensities 10 and 20 kV/cm. This signaled that the surface hydrophobicity of BSA dramatically reduced after glycation and it should be owing to the inhibited exposure of tryptophan residues to the solvent. This observation was similar to the one in a previous study [10]. In addition, lower *λ*_max_ and fluorescent intensity were revealed at the intensity of 20 kV/cm in both BSA–glucose and BSA–mannose compared with those treated at 10 kV/cm. This suggested a lower surface hydrophobicity in BSA–glucose and BSA–mannose prepared at the intensity of 20 kV/cm. Meanwhile, no significant differences were observed between BSA–glucose and BSA–mannose at the same PEF intensity.

### 2.4. Secondary Structure Analysis

Table 1 shows the changes in secondary structure. In the control test (B0), BSA had approximately 56.2% of α-helix, 7.6% of β-sheet, 10.8% of β-turns and 25.4% of random coil in the aqueous state. No significant changes were found in the blank tests (B1 and B2), suggesting that PEF treatment had no influence on the secondary structure of BSA at intensities 10 and 20 kV/cm. 

However, in the presence of glucose or mannose, the secondary structure changed significantly depending on the intensity. For example, the α-helix, β-sheet, β-turns and random coil were changed to approximately 45.3%, 9.6%, 13.9% and 31.2% after glycation with glucose at 10 kV/cm (G1). Moreover, more significant changes were found at the intensity of 20 kV/cm (G2) and the content of random coil reached 35.6%. Similar results were also found in BSA–mannose (M1 and M2). Overall, a decreased α-helix and increased random coil occurred in BSA after glycation with glucose or mannose. This was in accordance with previous reports, in which the BSA–glucose and BSA–mannose were prepared by other methods [10,28]. The lowest α-helix and highest random coil were found in M2. 

Electric field–dependent alterations were also reported in the literature, where BSA–dextran conjugates were obtained under similar conditions [20]. Besides, decreased α-helix and increased random coil in glycated BSA were ascribed to increasing intermolecular interaction among the neighboring proteins [20]. 

### 2.5. Emulsifying Activity

**Figure 4:**
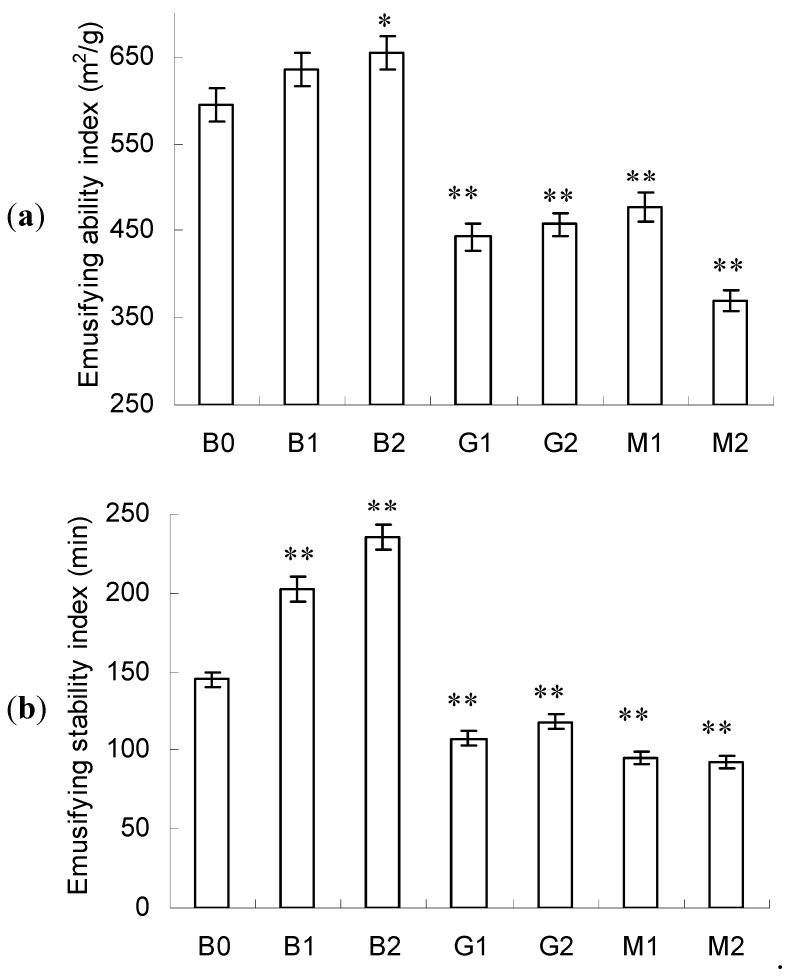
Emulsifying ability index (**a**) and emulsifying stability index (**b**) of BSA, BSA–glucose and BSA–mannose at intensities 10 and 20 kV/cm. Data are mean ± standard deviation, *n* = 3. * *p* < 0.05, ** *p* < 0.01 versus control (B0).

As shown in Figure 4a, no significant changes in the emulsifying ability index were found in B1 compared with control (B0) but a marked improvement was seen in B2 (*p* < 0.05). In contrast, the emulsifying ability index sharply decreased after glycation with glucose (G1 and G2) or mannose (M1 and M2) (*p* < 0.05). The lowest emulsifying ability was revealed in M2. Indistinctive differences in emulsifying ability index were observed between G1 and G2 but the emulsifying ability index of M2 was significantly lower than that of M1. 

A similar trend was also found in emulsifying stability index (Figure 4b). Markedly improved emulsifying stability index was found in B1 and B2 (*p* < 0.01) and significantly decreased emulsifying stability index was found in G1, G2, M1 and M2 (*p* < 0.01). No distinctive difference was found between M1 and M2. These observations were in accordance with the literature [29], that is, no improvements in emulsion stability were observed for BSA–sugar (glucose, allose and 6-*O*-octanoyl-d-glucose) conjugates. 

Based on the above analysis, PEF treatment favored the improvement in the emulsifying activity of BSA but glycation with glucose or mannose showed a negative effect. Such a phenomenon should be ascribed to the changes in surface hydrophobicity. Generally, increased surface hydrophobicity favored the improvement in emulsifying activity [30]. The increased emulsifying activity in B1 and B2 should be owing to the increased surface hydrophobicity, as indicated by their red-shifting *λ*_max_ in intrinsic fluorescence spectra (Figure 3). The increased exposure of hydrophobic groups to the solvent made it easier to absorb at the oil interface in oil/water system, resulting in a higher emulsifying ability. Conversely, the decreased emulsifying activity in G1, G2, M1 and M2 should be ascribed to the decrease in surface hydrophobicity, as depicted in Section 3.3.

### 2.6. Foaming Activity

FC and FS are two vital parameters of foaming activity. FC indicates the capacity of including the air into the continuous phase and the latter relates to the ability to retain the air for a given time. Both FC and FS are influenced by conformational flexibility, surface hydrophobicity and other factors [31].

**Figure 5:**
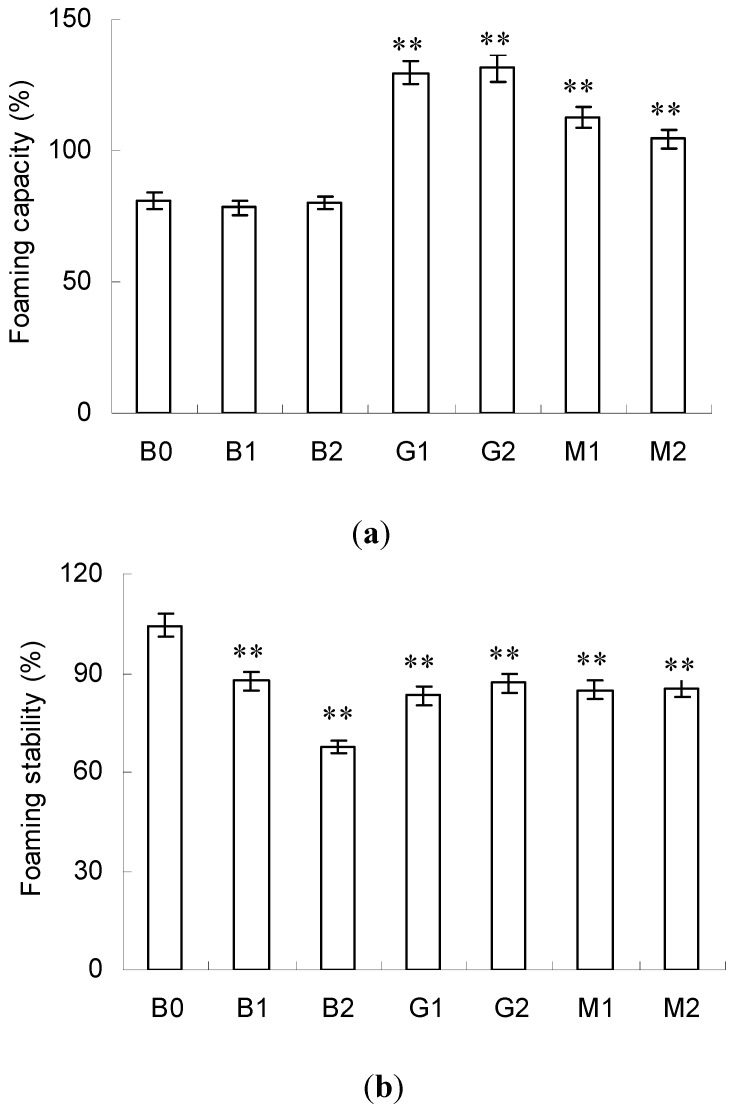
Foaming capacity (**a**) and foaming stability (**b**) of BSA, BSA–glucose and BSA–mannose at intensities 10 and 20 kV/cm. Data are mean ± standard deviation, *n* = 3. ** *p* < 0.01 versus control (B0).

As shown in Figure 5a, the FC of BSA remained unchanged in B1 and B2. However, significant improvement was found in G1, G2, M1 and M2 (*p* < 0.01) and the highest value was obtained in G1 and G2. Comparison of the surface hydrophobicity and secondary structure of BSA before and after PEF treatment indicated the aforementioned differences in FC might be ascribed to the secondary structure. Generally, loosened and flexible conformation favored FC, whereas compact and rigid conformation had a negative effect [32]. Thus, the increased FC should be due to the relatively loosened and flexible conformation in G1, G2, M1 and M2, as indicated by their higher content of random coil. 

Contrary to the changes in FC, markedly decreased FS was revealed in all samples compared with the control (B0). The lowest FC was found in B2. FS was synthetically affected by surface hydrophobicity, solubility and interfacial properties of proteins. In general, higher surface hydrophobicity meant higher affinity for the air/water interface and resulted in an increased FS. Therefore, the decrease in FS in G1, G2, M1 and M2 was mainly due to the decreased surface hydrophobicity, as indicated by their blue-shifting *λ*_max_ in Figure 3. However, this rule was not applicable in the case of B1 and B2 and the detailed mechanism should be further explored. 

Besides surface hydrophobicity and secondary structure, surface charge may be also a vital factor affecting foaming and emulsifying properties. As observed in literature, changed surface charge occurred in BSA during glycation due to the involvement of the basic amino groups in Maillard reaction [33]. Generally, the decreased surface charge caused a more rapid adsorption of protein at the air-water interface and increased dilatational viscoelasticity and foam yield stress [34]. Thus, the changed FC and FS in B1, B2, M1 and M2 may be partly ascribed to the changes of surface charge. Another, the changed surface charge may also play a part in the emulsifying properties. In general, surface charge of the protein not only influences the solubility of the proteins within the aqueous phase but also affects the electrostatic repulsion between oil droplets [30]. High electrostatic repulsion between oil droplets tends to lead to greater emulsion stability, whereas low electrostatic repulsion leading to coalescence and instability. Hence, the surface charge in B1, B2, M1 and M2 would be determined in future by 2D isoelectric focusing sodium dodecyl sulfate polyacrylamide gel electrophoresis (IEF/SDS-PAGE).

Recently, the progress in light scattering/diffraction techniques and microscopic approaches, such as multiple light scattering technique [35] and atomic force microscopy [36], offer a reliable choice to fully assess the emulsification and foaming capacity of a potential emulsifier. They could easily assess the size of droplet and even differentiate creaming, coalescence or Ostwald ripening. Whether they are suitable for this research or not would be fully studied in future research. 

## 3. Materials and Methods

### 3.1. Chemicals and PEF System

BSA, glucose and mannose were obtained from Sigma–Aldrich (St. Louis, MO, USA) and corn germ oil was purchased from the local market. All other chemicals were of analytical grade and obtained from Merck (Darmstadt, Germany).

A continuous PEF system was provided by Prof. Xi-an Zeng from the School of Food Science and Engineering, South China University of Technology (Guangzhou, China). The PEF system was mainly composed of a constant flow pump (323 E/D, Watson Marlow, Wilmington, MA, USA), a flow meter (LZB-4, Changzhou Shuangfa Thermal Instrument Factory, Changzhou, China), a digital oscilloscope (DST1102B, Tekway Technologies Co., Ltd., Nanjing, China) and the treatment chamber. The treatment chamber with a flow volume of 0.02 mL consisted of two parallel titanium-based alloy electrodes and a tubular insulator body made of Teflon. The gap of the electrodes in the treatment chamber was 0.30 cm. The temperature of sample solution in inlet and outlet was monitored by thermocouple. A cooling circulator (DLSK 3/10, Ketai Laboratory Equipment Co., Ltd., Zhengzhou, China) was used to control the temperature of the sample. The detailed configuration and operation procedure of the PEF system were according to those illustrated in the previous studies [20,37]

### 3.2. Preparation of BSA–Glucose and BSA–Mannose Conjugates

BSA–glucose and BSA–mannose conjugates were prepared as previously described with minor modification [22]. Normally, higher ratio of sugar/protein favored the glycation of protein. Based on previous study obtained by thermal treatment, 1.0 g BSA and 1.0 g glucose or mannose were added together and dissolved in 190 mL of deionized water. Then, the pH of the solution was adjusted to 10.0 with 6 M NaOH and the electrical conductivity of the solution was adjusted to 4.0 ± 0.1 ms/cm with NaCl solution. Afterward, the solution was replenished to a final volume of 200 mL with deionized water. As a contrast, the solution of BSA without glucose or mannose was prepared in parallel with the aforementioned mixture solutions. All solutions were stored at 4 °C in a refrigerator before and after PEF treatments. 

The solutions were subjected to PEF treatment using the following operating parameters. A unipolar square wave was used with 1-kHz pulse frequency, 20-μs pulse duration and 73.5 pulses. The flow rate of the solution was 30 mL/min. For one circle treatment, the time of the solution subjected to PEF was 1.47 ms, which was calculated using pulse number and pulse width. A total of 12 circle treatments were performed for a single solution. Therefore, the total PEF treatment time was 14.7 ms. The intensity of PEF was set as 10 and 20 kV/cm, by adjusting the voltage from 0.15 to 0.3 kV. After each circle of PEF treatment, the solution was cooled with cold water (4 °C) for 5 s to eliminate the thermal accumulation induced by the treatment. Then, the treated solutions were instantly cooled to 23.0 ± 2 °C with ice-cold water (4 °C) and dialyzed in deionized water (room temperature, 18 MΩ) for 48 h to remove the remaining monosaccharides and other resultant small molecules. After dialysis, the obtained solution was freeze-dried and used for subsequent experiments.

For convenience, the obtained BSA and its resultant conjugates were named by the combination of raw materials and the intensity of PEF. BSA without PEF treatments was named as B0 (control). BSA without glucose or mannose treated with PEF at the intensity of 10 or 20 kV/cm was named as B1 or B2, respectively. Similarly, BSA–glucose and BSA–mannose conjugates produced by PEF at the intensity of 10 kV/cm and 20 kV/cm were named as G1 or G2 and M1 or M2, respectively.

### 3.3. Measurement of Intermediate Products and Browning Value

The content of intermediate products and browning value in Maillard reaction products were measured using absorbance at 294 and 420 nm, respectively, using an Agilent Cary 60 UV-Vis spectrophotometer (Agilent, Santa Clara, CA, USA). Before measurement at 294 nm, a 20-fold dilution was made using distilled water [10].

### 3.4. Determination of Conjugation Ratio in Glycated-BSA Conjugates

In a previous study, the conjugation ratio in glycated-BSA conjugates was determined by calculating the decreased number of free amino groups in BSA after glycation using the 2,4,6-trinitrobenzenesulfonic acid (TNBS) assay [10].

Briefly, the determination process was as follows. First, 0.5 mL of BSA or glycated-BSA solutions with different concentrations (0, 0.2, 0.4, 0.6, 0.8 and 1.0 mg/mL) were prepared using the solution of chloride sodium (0.9%, *w*/*v*). Then, they were mixed with 0.5 mL of carbonate buffer (0.2 mol/L, pH 9.6) and 0.5 mL of TNBS solution (0.1%, *w*/*v*) and placed in the dark for 15 min at room temperature. Finally, the absorbance of the mixture at 420 nm (A_420_) was determined using Agilent Cary 60 UV-Vis spectrophotometer (Agilent, Santa Clara, CA, USA). Afterward, the slope coefficient (*k*) of BSA or glycated-BSA was calculated using the slope of A_420_ versus concentration and the conjugation ratio of glycated-BSA was determined using an equation reported in a previous study [38].

### 3.5. Measurement of Surface Hydrophobicity 

According to a previous study [39], the surface hydrophobicity was characterized by the fluorescent intensity of the solution. The protein samples were dissolved in phosphate buffer (10 mM, pH 7.0) and then the fluorescence spectroscopy was performed using a spectrofluorometer (FL, Hitachi, Japan) in the range of 300–600 nm using phosphate buffer as the blank. The excitation wavelength and slit width were set at 279 and 5 nm, respectively.

### 3.6. Circular Dichroism Analysis

The far-ultraviolet circular dichroism (CD) analysis was performed using a Chirascan Circular Dichroism Spectrometer (Applied Photophysics, Surrey, UK) in the range of 190–250 nm. The protein samples were dissolved in the phosphate buffer (10 mM, pH 7.0) and the phosphate buffer was used as the blank. The bandwidth was set at 0.5 nm and the integration time was set as 1 s. Consequently, the CD spectrum was analyzed using the SELCON3 software (Applied Photophysics, Surrey, UK) [27].

### 3.7. Assay on Physicochemical Properties

The emulsifying activity index (EAI) and emulsion stability index (ESI) were determined according to the method developed by Tang [26]. The EAI and ESI were calculated based on the absorbance obtained at 500 nm after mixing the samples with corn oil and homogenization. The detailed determination method was described as follows. First, protein solution (15 mL, 1.0% *w*/*v*) and corn oil (5 mL) were mixed and homogenized for 1 min at 20,000 rpm using Ultra-Turrax T25 (IKA Co., Staufe, Germany). Instantly, 2 mL emulsion was taken from the bottom of the container and diluted (1:50, *v*/*v*) by SDS solution (0.1%, *w*/*v*). Then, the absorbance of diluted emulsion at 500 nm was measured with a spectrophotometer. Same operation was done 10 min later after homogenization. Finally, EAI and ESI were calculated using equation, which was fully depicted in a previous study [10].

According to the previous study [40], the foaming capacity (FC) and foaming stability (FS) were measured after dissolving the samples in phosphate buffer and homogenization. The detailed process was reported as follows. Protein solution (20 mL, 0.5% *w*/*v*) in phosphate buffer (10 mM, pH 7.0) was homogenized in a homogenizer (Ultra-Turrax T25, IKA Co., Staufe, Germany) for 1 min at 20,000 rpm. After homogenization, the volume of the solution was measured immediately and 30 min later respectively. Then, FC and FS were calculated respectively using equation fully described in a previous study [10].

### 3.8. Statistical Analysis

All the experiments were performed in triplicate and the results were expressed as mean ± standard deviation. Analysis of variance was conducted in GraphPad Prism 5 using the Tukey test method. A *p* value < 0.05 was considered significantly different.

## 4. Conclusions

The present study showed that PEF treatment was an effective method to prepare BSA–glucose and BSA–mannose conjugates under mild conditions. PEF treatment not only promoted Maillard reaction between BSA and glucose or mannose but also alleviated the undesirable browning observed in the traditional thermal treatment. PEF treatment favored the increased surface hydrophobicity and emulsifying activity in BSA and had no effect on its secondary structure and FC. On the contrary, glycation with glucose or mannose reduced surface hydrophobicity and FS and improved FC. These changes in foaming and emulsifying activities of BSA were highly related to its surface hydrophobicity and secondary structure. These findings provided useful insights into the application of PEF treatment as a potential method to prepare BSA–glucose and BSA–mannose conjugates by Maillard reaction.

## Figures and Tables

**Table 1:** Secondary structure composition of BSA, BSA–glucose and BSA–mannose at intensities 10 and 20 kV/cm.

	α-Helix (%)	β-Sheet (%)	β-Turns (%)	Random Coil (%)
B0	56.2 ± 0.3	7.6 ± 0.4	10.8 ± 0.2	25.4 ± 0.3
B1	55.0 ± 0.4	7.3 ± 0.5	11.3 ± 0.3	26.4 ± 0.2
B2	57.4 ± 0.3	7.4 ± 0.3	9.6 ± 0.4 *	25.6 ± 0.2
G1	45.3 ± 0.4 **	9.6 ± 0.3 *	13.9 ± 0.2 *	31.2 ± 0.4 *
G2	42.8 ± 0.3 **	10.2 ± 0.3 *	11.4 ± 0.4 *	35.6± 0.4 *
M1	43.2 ± 0.5 **	8.9 ± 0.4 *	12.0 ± 0.3 *	35.9 ± 0.3 *
M2	41.6 ± 0.2 **	9.5 ± 0.3 *	12.5 ± 0.4 *	36.4 ± 0.3 *

Data are mean ± standard deviation, *n* = 3. * *p* < 0.05, ** *p* < 0.01 versus control (B0).
